# Supplementary material for: Impact of industrial robot applications on global value chain participation of China manufacturing industry: Mediation effect based on product upgrading
Source: PLoS One. 2023 Nov 8;18(11):e0293399. doi: 10.1371/journal.pone.0293399 (PMC10631699; doi:10.1371/journal.pone.0293399)
Supplement: Attachment — Submitted filename: Response to Reviewers.docx [file pone.0293399.s001.docx]

**Response to** **Reviewers**

Thank you very much to Professor Ali and the reviewers for giving me this valuable opportunity to revise my paper. After a long time of effort, I have finally completed this revision. Now, I will provide the following explanation for the revisions. If there are any mistakes, please continue to provide criticism and assistance from all experts. We appreciate it very much.

**1.****Response to the academic editor**

**（1）GVC should be an in percentage. The authors are advised to divide the actual GVC by the gross export (GVC participation is equal to the sum of forward and backward participation, divided by the gross export). then run the regressions again.**

Thank you very much for your valuable feedback. Based on your feedback, I have re examined the variable settings in this article. This paper measures the GVCP of Chinese industries using the GVCP measure proposed by Koopman et al., as shown in Equation (1).

$GVCP=\frac{IV}{E}+\frac{FV}{E}$	(1)

In Equation (1), GVCP is the global value chain participation index; IV denotes the domestic value-added in intermediate products processed in the importing country and then exported to third-party countries; FV denotes the foreign value-added in domestic exports, and E denotes total exports. The larger the index is, the higher is the industry's participation in GVCs. Further, this participation index can be decomposed into forward and backward participation, with IV/E being forward participation and FV/E being backward participation. Forward and backward participation can indicate the degree of participation of a country's industry in the upstream and downstream links of GVCs.

In summary, the calculation method of GVCP is in line with your opinion. This article supplemented the control variables based on the review comments and re conducted regression analysis in sequence.

**（2）The differences between industries with different technological levels should be controlled.**

Thank you very much for your valuable feedback. This article uses binary dummy variables to distinguish between high and low tech manufacturing industries, where "1" represents high-tech manufacturing and "0" represents low-tech manufacturing. To test whether the basic regression results will be affected by different technological levels in the manufacturing industry, this article introduces moderating variables for regression analysis. By setting the intersection term between IR applications and high-tech/low-tech manufacturing virtual variables as the explanatory variable, basic equations is regressed again. The empirical results indicate that IR applications have a significant inhibitory effect on the forward and backward GVCP of China's manufacturing industry in both high-tech and low-tech industries.

**（3）Use the following recent articles to strengthen the background of your paper.**

Thank you very much for your valuable feedback. After carefully reading these academic papers, we have added them to the statement of research background (including literature review and research hypotheses) accordingly.

**2.Response to the reviewer 1**

**（1）The contribution of the paper is unclear. You need to improve the contribution paragraph.**

Based on your valuable feedback, we have rewritten the research contribution section.

**（2）You state that “In Equation (1), i and t represent manufacturing industry segment and year, respectively; GVCP is global value chain participation, which is the explanatory variable;…..”. What is the dependent variable is in this equation? You need to clarify this.**

Based on your valuable feedback, we have made corresponding modifications to this section: ‘In Equation (1), i and t represent manufacturing industry segment and year, respectively; GVCP is global value chain participation, which is the result variable; IR represents the degree of IR applications, which is the explanatory variable.’

**（3）You try to describe data, but this it’s not enough. What are the units of the data you use?**

Based on your valuable feedback, we have made corresponding modifications to the data description section and added annotations to Table 1. ‘Note: ①The unit of ‘robot stock’ is one robot, for example, the value of 0.01 in the food and beverages industry in 2006 represents a robot inventory of 0.01 units per 1,000 employed persons in2006. The larger the number, the higher the level of IR applications in the industry. ②The number range of GVCP is from 0 to 1, and there is no specific unit. The larger the number, the higher the GVCP representing the industry.’

**（4）Estimation technique. First, what is the estimation technique used for the findings reported in Tables 2-3 and Table 5? This is important because a section on estimation technique is missing. Second, throughout the text, we realize that you are using the instrumental variables (IV) approach and this is a good way to deal with the endogeneity issue. However, which type of the IV approach are you using? GMM, 2SLS or 2SLS+FE/RE? This is not clearly mentioned in the first paragraph of the section 5.3.2. Third, you state “There are two main sources of endogeneity:….” This statement is not correct. You should heck further.**

Thank you very much for your valuable feedback. We have made the following revisions in sequence.

First, the estimation technique used in Table 2, Table 3 and Table 5 (now Table 7) is Ordinary least squares (OLS).

Secondly, the estimation technique used in Table 4 is the two-stage least squares method (2SLS), which controls for time and industry fixed effects.

Finally, we have made revisions to the discussion on the reasons for endogeneity, not limited to the two reasons proposed in the original manuscript.

**（5）Diagnostic tests. Some diagnostic tests** **(such as correlation and multicollinearity tests and unit-root test) are needed to support your findings. This is important because, if variables are not stationary at level, other techniques will be useful rather than the IV approach. Also, the inclusion of year fixed effect can capture the effect of some observable variables. Kindly refer some papers like https://doi.org/10.1007/s10644-023-09503-x ; https://doi.org/10.1111/ecot.12303.**

Thank you very much for your valuable feedback. We have added corresponding diagnostic test instructions (such as correlation and multicollinearity tests and unit root tests) in the descriptive analysis section.

(i) Through the calculation of Pearson correlation coefficient, it was found that there were no values exceeding 0.40 in the three samples of GVCP, forward GVCP, and back GVCP, indicating a weak correlation between the variables.

(ii) Through the calculation of the VIF coefficient, it is found that there is no more than 2.00 values in GVCP, forward GVCP, and backward GVCP, which indicates that there is no multicollinearity problem between variables.

(iii) This paper conducts LLC test on the panel unit-root of GVCP, forward GVCP, and backward GVCP, and finds that these adjusted $t_{\delta}^{*}$ statistics are significantly negative, which means strongly rejecting the original assumption that the panel contains Root of unity and accepting the conclusion that the panel is a Stationary process. Further, after subtracting the cross-sectional mean from Panel data, the LLC test was conducted to avoid the possible cross-sectional correlation effects of the disturbance terms in different industries. The results showed that these adjusted $t_{\delta}^{*}$ statistics are still significantly negative at the 1% level.

Finally, based on the literature recommended by the reviewers, important information that may be implied by the fixed time effect was discussed, and relevant references were added to the references.

**（6）Tables of results. It is difficult to understand your Tables. Why manufacturing robot stocks in US appear in Table 4 while this specification is missing in previous Table 2 or 3. According to me you applied traditional techniques in Table 2 (or3) and in Table 4 you applied the IV approach to deal with the endogeneity issue. If it is a case, I think that your explanatory variable of interest (IR) must be the same when you move from Table 2 (or 3) to Table 4. In the same vein, for Table 5, you must present the findings based on traditional and IV approach methods.**

Thank you very much for your valuable comments. The number of robots stocked per 1000 employed population in the related industries in the United States is taken as a Instrumental variables estimation in the columns (1) to (3) of Table 4, and the regression analysis of equation (1) is conducted again using the 2SLS method. Columns (4) to (6) of Table 4 use the lag phase I of the explanatory variable as a Instrumental variables estimation, and use OLS method to conduct regression analysis on equation (1) again. Table 5 (now Table 7) follows the step by step regression steps of Mesomeric effect, and tests the Mesomeric effect of product upgrading. OLS technology is used for regression analysis. However, whether the stepwise regression analysis using the 2SLS method can test the Mesomeric effect, the relevant literature we have (for example, MacKinnon et al. [51], Selig and Preacher [52], and Zhao et al. [53]) does not carry out a robustness test on the Mesomeric effect. So, we are currently unable to solve this issue effectively. Please understand and thank you very much.

**（7）Presentation of the empirical results and their discussion. The Author has separated Section 5 Empirical result and analysis from Section 6 Research findings and discussion. This manner of presentation is less recommended. According to me, Section 5 does not differ from Section 6. In the same vein, it is be useful to report the empirical result and analysis and research findings and discussion in the same section. Also, the decomposition of the Section 6 into two sub-sections 6.1 research findings and 6.2 research discussion is not necessary, this means that the findings and their discussion must be simultaneously presented.**

Thank you very much for your valuable feedback. We have made significant deletions to the last part of the original manuscript and have rewritten it.

**（8）Conclusion. The whole conclusion section is ignored. I recommend the author to include a conclusion section.**

Thank you very much for your valuable feedback. We have rewritten the conclusion section of this article based on the modification suggestions mentioned in (7) above.

**（9）Language editing. The current version of the paper is not well edited. Grammar and spelling checks are required.**

Thank you very much for your valuable feedback. We have selected PLOS ONE's language polishing service to fully revise the manuscript language.

**3.Response to the reviewer 2**

**（1）Section 4.2.1 spend a lot of space to introduce the background of the value-added decomposition framework. This seems unnecessary.**

Thank you very much for your valuable feedback. There are many methods for measuring GVCP, and we tend to measure this variable from the perspective of value-added decomposition. Therefore, we have devoted more space to explaining the calculation of GVCP.

**（2）The author does not consider the differences between industries with different technological levels.**

Thank you very much for your valuable feedback. We have added the 'Analysis of Technical Heterogeneity' section to analyze the technical heterogeneity of the basic regression results.

**（3）Compared to product upgrading, production costs and production efficiency also play an important role in the impact of IR application on GVC participation. However, this is not analyzed in this paper.**

Thank you very much for your valuable feedback. As this article mainly aims to illustrate that product upgrading is an important factor affecting the decline of the global value chain in China's manufacturing industry, it mainly focuses on product upgrading for discussion. Based on your revision comments, we have provided a theoretical explanation of the other variables that may have an impact that you proposed in the literature review section.

**（4）It is suggested that the author revise the title of Section 4.1.1 and Section 4.1.2 to highlight the research content. The same problem also exists in Section 5.**

Thank you very much for your valuable feedback. We have made modifications to the corresponding titles.

**4.Response to the reviewer 3**

**（1）The literature review is comprehensive enough and discuss the constituents of the GVC participation and industrial robots nexus deeply. However, there are too much repetition in the paper. For example, the concept of "make cage for bird" is repeated three times in the paper. To avoid the repetitive arguments and phrases throughout the manuscript, Sections 3 and 4 can be merged into more shortened section presenting hypotheses and a model.**

Thank you very much for your valuable feedback. We have edited and improved the literature review and theoretical assumptions section of the manuscript.

**（2）The conclusion that the negative impact of IR applications on GVC participation could possibly be related to the size and development levels of a country cannot be generalized and applied to other cases though because the results are presented here come from Chinese economy. This limitation should be mentioned in the text.**

Thank you very much for your valuable feedback. We have explained the limitations of the research in the abstract, introduction, research conclusion, and other parts of the manuscript. The research conclusion of the manuscript is only applicable to the Chinese sample.

**（3）GVC participation can be calculated by different methodologies such as Koopman et al (2010) (trade based) and Wang et al (2017) (production based). Here the author chooses the methodology of Koopman et al (2010). The alternative measures can be mentioned as further research directions.**

Thank you very much for your valuable feedback. As the data we currently have mainly involves trade value-added, if we have suitable data in the future, we will try to calculate GVCP from the perspective of production, in order to obtain some valuable research conclusions.

**（4）This study covers the period 2006-2014 regarding the WIOD. However, OECD TİVA 2021 continue until 2018 and OECD TiVA 2022 continues until 2020. This remaining period from 2014 to 2020 includes very significant worldwide occasions having rigorous impact on GVCs. The author may clarify that the availability of the Socio Economic Accounts (SEA) of the WIOD is superior to OECD TiVA Data especially in terms of the availability of the control variables.**

Thank you very much for your valuable feedback. As the 2016 version of WIOD is currently the latest publicly available data, we regret to set the empirical period to 2006-2014. Although we are currently unable to address the issue of data timeliness, the research conclusion is quite relevant to the Chinese context, that is, the global value chain of China's manufacturing industry is indeed showing a downward trend, which is consistent with the transfer of labor-intensive industries from the eastern coastal areas of China to the central and western regions, as well as the current construction of domestic circular markets. In the future, on the one hand, we actively collect the latest data for regression analysis, and on the other hand, through Case study of Chinese enterprises, we hope to further expand this study. I hope our answer can receive your understanding. Thank you very much.

**（5）The author seems to largely ignore the role of foreign direct investment and multinationals in the discussions. The author can provide some discussion on this.**

Thank you very much for your valuable feedback. Due to data limitations, we cannot include variables such as foreign direct investment in the control variables. But based on your opinion, we have added relevant literature to the literature review and theoretical hypothesis section for theoretical explanation.

**（6）I recommend author to control the impact of the 2008 global economic crisis by employing a dummy variable.**

Based on your revision comments, we have added dummy variables from the 2008 financial crisis to the control variables and conducted a new regression analysis.

**（7）Table 1 should also present the data on backward and forward GVCP individually to the impact on each of them.**

Based on your revision comments, we have added data descriptions of forward and backward participation in the global value chain of China's manufacturing industry in Table 1. Thank you very much for your valuable feedback.

**（8）As robustness checks, in addition to the use of instrumental variable of IR in the United States, the author may also provide the estimates with IR in the EU and Japan.**

Thank you very much for your valuable comments. In the Instrumental variables estimation section of the manuscript, the reason for using US data is explained, that is, US data meets exogenous and relevance requirements, and is an ideal Instrumental variables estimation. Considering that China, along with India, Brazil, South Africa, and Russia, are emerging economies with faster development rates, corresponding regression analysis can be conducted using data from these countries, and the basic conclusions have been verified. Due to some missing data from South Africa, we only returned data from India, Brazil, and Russia, as shown in the table below. The research results indicate that the application of industrial robots significantly suppressed the overall and backward participation in the global value chain of manufacturing in these developing countries. Although the forward participation did not pass the significance test, the regression coefficient was negative.

Table1 Based on regression results for India, Brazil, and Russia

|  | (1)  GVCP | (2)  Forward GVCP | (3)  Backward GVCP |
| --- | --- | --- | --- |
| IR | -0.0089*** | -0.0009 | -0.0080*** |
|  | (0.0019) | (0.0018) | (0.0022) |
| CTR | YES | YES | YES |
| Ind FE | YES | YES | YES |
| Year FE | YES | YES | YES |
| Country FE | YES | YES | YES |
| R-squared | 0.7592 | 0.8394 | 0.6210 |
| Observations | 570 | 570 | 570 |

Note: ∗∗∗p < 0.01, ∗∗p < 0.05, and ∗p < 0.1; Robust standard error in parentheses.
