# Supplementary material for: Impact of industrial robot applications on global value chain participation of China manufacturing industry: Mediation effect based on product upgrading
Source: PLoS One. 2023 Nov 8;18(11):e0293399. doi: 10.1371/journal.pone.0293399 (PMC10631699; doi:10.1371/journal.pone.0293399)
Supplement: Attachment — Submitted filename: Response to Reviewers.docx [file pone.0293399.s002.docx]

**Response to** **Reviewers**

Thank you very much to Professor Ali and the reviewers for giving me this valuable opportunity to revise this paper. Now, we will provide the following explanation for the revisions. If there are any mistakes, we sincerely hope to continue to receive guidance from Professor Ali and the reviewers.

Kind regards,

Shuangzhi Zhang

***Data Availability Statement****: All relevant data are within the manuscript and its Supporting information files.*

*The dataset of this paper has been uploaded as an attachment in the submission system and has been uploaded to the Dryad database.*

*DOI: 10.5061/dryad.280gb5mvr*

*URL: https://datadryad.org/stash/share/ojZHg4V2fxznQ402yT_jo6EkP4fFblF2co_d5iI0VJM.*

**1.****Response to the Academic Editor**

**（1）Please take seriously the concerns raised by reviewer 1. I Checked the paper and, fully agreed with him.**

Thank you very much for your valuable feedback. After carefully reading the comments of Reviewer 1, we made careful revisions one by one. The specific situation can be seen in the response to Reviewer 1's comments.

**（2）The following paper can strengthen your research background.**

Thank you very much for providing these important references. After carefully reading this paper, we will use it to expand the content of the literature review. We have added it as reference [33].

**2.Response to the Reviewer 1**

**（1）The author has discussed some diagnostic tests as previously requested. However, this discussion needs to be supported by some previous studies. For some of these tests, again, kindly refer to some recent papers like https://doi.org/10.1007/s10644-023-09503-x ; https://doi.org/10.1016/j.strueco.2022.10.003 ; https://doi.org/10.1111/ecot.12303.**

Thank you very much for providing these important references. After carefully reading these papers, we will use them as an important basis for the research background and empirical model selection of this paper. We have added them as references [61], [65] and [67].

**（2）Through the text, some abbreviations, like LLC, need to be defined first.**

Thank you very much for your constructive feedback. Based on your valuable feedback, we have provided an explanation and full name description of the first appearance of LLC. We have also carefully reviewed the entire text to avoid similar errors.

**（3）The conclusion section must be revised.**

**(i) Some important points like the methodology applied and the main findings should be provided in the conclusion.**

**(ii) Policy implications should flow directly from your findings.**

**(iii) Each study has its strengths and weaknesses (limitations). Thus, the author should provide avenues for future research.**

Based on your valuable feedback, we have rewritten the conclusion section, which is very important for improving the quality of this paper. The specific content is as follows:

This paper uses data from 13 manufacturing industries in China from 2006 to 2014 to empirically test the impact of IR applications on forward and backward participation in the GVCs of China's manufacturing industry. The regression results of the two-way fixed effects model indicate that IR applications lead to a simultaneous decrease in forward and backward participation in the GVCs of China's manufacturing industry. Using the 2SLS model to perform endogeneity tests on the basic regression results, it was found that the regression results of the two-way fixed effects model has good robustness. Furthermore, using the moderating effect model, the technology heterogeneity of manufacturing industry was included in the regression analysis. The empirical results showed that the application of IR suppressed both forward and backward participation in the GVCs of China's manufacturing industry, whether in high-tech or low-tech industries.

Based on the mediation effect model, this paper explains the reasons for the decline of China's manufacturing GVCP caused by IR applications from the perspective of product upgrading. IR applications promote product upgrading of China's manufacturing industry, making China more inclined to retrieve or retain manufacturing. This may have two impacts: (i) Product upgrading can help China achieve the import substitution of intermediate inputs and use local intermediate inputs to produce exports. This will reduce backward participation in the GVCs of China's manufacturing industry. (ii) Localization of manufacturing can make an economy. However, the localization of manufacturing can cause China to lose the opportunity to export intermediate inputs to other economies, thereby reducing forward participation in the GVCs.

Based on empirical research results, this paper proposes the following policy recommendations.

(i) Although the current application of IR in China has not promoted forward participation in the GVCs, the experience of developed countries shows that IR applications can help achieve an increase in forward participation. This means that China needs to strengthen the research and development of AI technology and tackle key problems, and fully leverage the empowering effect of "IR+" with a scenario driven approach to produce new products with international competitive advantages, promote China's deep integration into the GVCs through participatory approaches.

(ii) The application of IR did not promote backward participation in the GVCs of China's manufacturing industry, which may be related to the obvious hierarchical characteristics of regional economic development within China. China needs to continue to promote the digital and intelligent transformation of its manufacturing industry, continuously enhance the added value of production and manufacturing links, promote the gradient transfer of production and manufacturing between different regions in the country, and more actively undertake the production and manufacturing activities of multinational enterprises.

(iii) The inhibitory effect of IR applications on forward and backward participation in the GVCs of China's manufacturing industry may be related to the shortage of highly skilled workers in the Chinese labor market. 'Machine substitution' does not mean that workers are no longer important, but rather that the demand for labor skills is increasing. Germany's development experience shows that highly skilled workers are an important advantage for 'Made in Germany' to participate in international competition. The Chinese government should actively learn from Germany's vocational education system and accelerate the adjustment of labor force to adapt to the changes brought about by the application of IR, which may promote the participation of China's manufacturing industry in the GVCs to move upwards.

Other avenues of research are possible. Subsequent research will adopt case study method, selecting several typical Chinese manufacturing enterprises as cases, and exploring the deep-seated reasons why the application of IR has led to a decrease in both forward and backward participation in the GVCs of Chinese manufacturing from two dimensions of theoretical construction and theoretical testing, in order to further enrich empirical research conclusions.

**3.Response to the Reviewer 3**

**It is suggested that the review includes the theoretical underpinnings linking the adoption of industrial robotics to the emergence of new skills and job profiles. The adoption of robotics may lead to the creation of new job profiles/skills, like robot maintenance or programming, while simultaneously reducing demand for low-skilled manual jobs. Countries that can quickly adapt their workforce to these changes can potentially move up the GVC ladder. Including these theoretical insights would enhance the depth of the review.**

Thank you very much for your valuable feedback. Your opinion provides a very important perspective for improving the argumentation height of this paper. Based on your opinion, we have attempted to supplement the conclusion section, and the specific content is as follows.

The inhibitory effect of IR applications on forward and backward participation in the GVCs of China's manufacturing industry may be related to the shortage of highly skilled workers in the Chinese labor market. 'Machine substitution' does not mean that workers are no longer important, but rather that the demand for labor skills is increasing. Germany's development experience shows that highly skilled workers are an important advantage for 'Made in Germany' to participate in international competition. The Chinese government should actively learn from Germany's vocational education system and accelerate the adjustment of labor force to adapt to the changes brought about by the application of IR, which may promote the participation of China's manufacturing industry in the GVCs to move upwards.
