# Supplementary material for: Impact of industrial robot applications on global value chain participation of China manufacturing industry: Mediation effect based on product upgrading
Source: PLoS One. 2023 Nov 8;18(11):e0293399. doi: 10.1371/journal.pone.0293399 (PMC10631699; doi:10.1371/journal.pone.0293399)
Supplement: Attachment — Submitted filename: Response to Reviewers.docx [file pone.0293399.s003.docx]

**Response to** **Reviewers**

**Dear Professor Ali and Reviewers:**

Thank you very much to Professor Ali and the reviewers for recognizing the revision work of this paper, which is very important to me. It is precisely based on your constructive suggestions that this paper can have a significant improvement in quality. Based on the suggestions of the reviewers and the journal guidelines, I have made very detailed revisions to the references in this paper and marked them in the text.

I would like to express my great appreciation to you and the reviewers for their comments on our paper. I look forward to hearing from you.

Thank you, and best regards.

Yours sincerely,

ShuangZhi Zhang

***Data Availability Statement****:* *All relevant data are within the manuscript and its Supporting information files. The dataset of this paper has been uploaded as an attachment in the submission system and has been uploaded to the Dryad database.*

*DOI: 10.5061/dryad.280gb5mvr*

*URL:* [*https://datadryad.org/stash/share/ojZHg4V2fxznQ402yT_jo6EkP4fFblF2co_d5iI0VJM*](https://datadryad.org/stash/share/ojZHg4V2fxznQ402yT_jo6EkP4fFblF2co_d5iI0VJM)

***Figure Files:*** *According to the journal's paper submission guidelines, all charts in this article have been uploaded to the Preflight Analysis and Conversion Engine (PACE) platform.*

**1.****Response to the Academic Editor**

**Kindly review the journal guidline and check the references styles. Please use the APA style for all references cited in the reference list.**

*Thank you very much for your valuable feedback. According to the journal's paper submission guidelines, I have made very detailed revisions to the references for this paper and marked them in the text. Please refer to the revised references, I hope that you will be satisfied with my revision.*

**2.Response to the Reviewer #1**

**Congratulations, the author(s) has(ve) fully taken into account our previous comments.**

**As additional comment, you should harmonize your references and respect the order these references. For example, the author(s) should review certain references that are incorrectly entered, particularly references beginning with Essotanam or Essossinam (examples are [8], [32], [33], [61], [65], [67]). Therefore, in the section of references, the first names of the authors must be abbreviated.**

*Thank you very much for your recognition of the revision work of this paper,* *which is very important to me. Based on your constructive feedback and journal guidelines, I have carefully revised the references for this paper. Please refer to the revised references, I hope that you will be satisfied with my revision.*
